# Probing the folding pathway of a consensus serpin using single tryptophan mutants

**DOI:** 10.1038/s41598-018-19567-9

**Published:** 2018-02-01

**Authors:** Li Yang, James A. Irving, Weiwen Dai, Marie-Isabel Aguilar, Stephen P. Bottomley

**Affiliations:** 1 Department of Biochemistry and Biomedicine Discovery Institute, Monash University,, Clayton, Victoria 3800 Australia; 2 UCL Respiratory and the Institute of Structural and Molecular Biology, University College London, London, United Kingdom

## Abstract

Conserpin is an engineered protein that represents the consensus of a sequence alignment of eukaryotic serpins: protease inhibitors typified by a metastable native state and a structurally well-conserved scaffold. Previously, this protein has been found to adopt a native inhibitory conformation, possess an atypical reversible folding pathway and exhibit pronounced resistance to inactivation. Here we have designed a version of conserpin, cAT, with the inhibitory specificity of α_1_-antitrypsin, and generated single-tryptophan variants to probe its folding pathway in more detail. cAT exhibited similar thermal stability to the parental protein, an inactivation associated with oligomerisation rather a transition to the latent conformation, and a native state with pronounced kinetic stability. The tryptophan variants reveal the unfolding intermediate ensemble to consist of an intact helix H, a distorted helix F and ‘breach’ region structurally similar to that of a mesophilic serpin intermediate. A combination of intrinsic fluorescence, circular dichroism, and analytical gel filtration provide insight into a highly cooperative folding pathway with concerted changes in secondary and tertiary structure, which minimises the accumulation of two directly-observed aggregation-prone intermediate species. This functional conserpin variant represents a basis for further studies of the relationship between structure and stability in the serpin superfamily.

## Introduction

**Figure 1:**
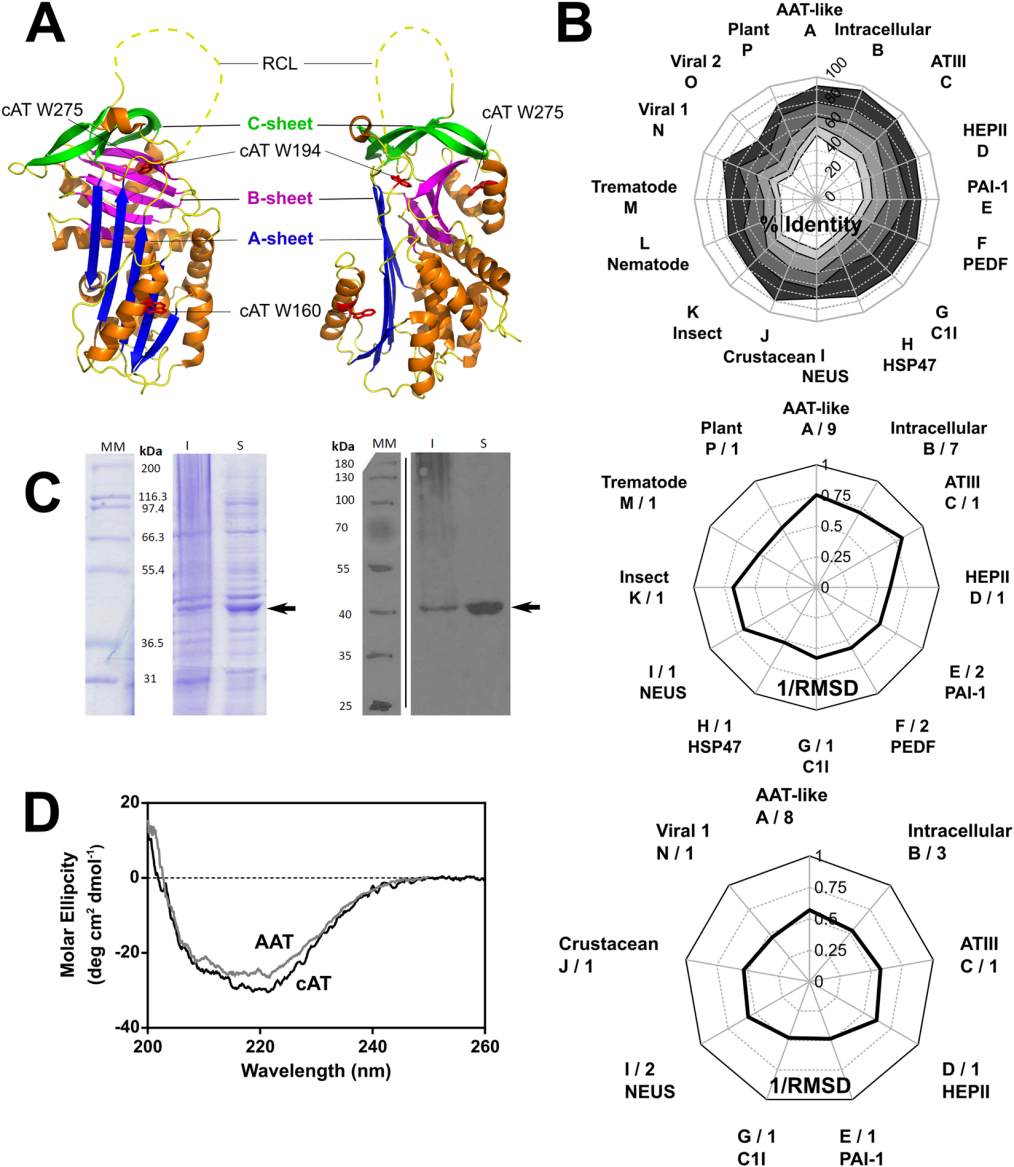
Properties of a conserpin variant. (**A**) The serpin fold is comprised of three β-sheets and eight or nine α-helices, illustrated using a cartoon representation of native conserpin (PDB 5CDX)^34^. Connecting β-strand 5 A and β-strand 1 C is the solvent exposed reactive centre loop (RCL) which dictates the target protease specificity of inhibitory serpins (unresolved in the structure and therefore denoted by a dashed line). Upon proteolytic cleavage, the RCL becomes a 6^th^ central strand of the normally 5-stranded β-sheet **A**. The positions of the tryptophan residues mutated in this study are indicated. (**B**) *Upper panel*: Radial plot of the average sequence identity between the consensus sequence and members of individual serpin phylogenetic clades^1^, calculated using all alignment positions (white central area), or the 75%, 50%, 25% and 10% lowest variability sites (shaded from light grey to dark grey). *Middle panel*: Reciprocal RMSD of native conserpin (PDB 5CDX) compared with the common core of representative structures from different phylogenetic clades. *Lower panel*: A comparison between latent conserpin (PDB 5CDZ) and representative cleaved/latent structures. (**C**) *Left*: SDS-PAGE (10% w/v, see full gels in Supplementary Fig. S1) section of cAT after 20 hours of auto-induction, visualised using Coomassie Blue stain. *Right*: Western blot of the corresponding SDS-PAGE, developed using an anti-His_6_ antibody. Lane MM, molecular weight markers; I, insoluble fraction; S, soluble lysate fraction. cAT is indicated by the arrow. (*D*) Far-UV spectrum of cAT (black) compared with α_1_-AT (grey) at a concentration of 0.2 mg mL^−1^, using a path length of 0.1 cm.

The serpin superfamily, with members in every phylogenetic kingdom, underwent a marked expansion in diversity following the plant-animal split^1–3^. The majority of serpins inhibit serine proteases, although some have been found to target classes of cysteine protease^4–6^ or perform alternative roles such as hormone transport, as chaperones and as tumour suppressors^7,8^. In contrast to most proteins^9^, the serpin native conformation is a kinetically-trapped, thermodynamically metastable folding intermediate. This characteristic is intimately associated with the mechanism of inhibition^10^, the essence of which is a transition of a central 5-stranded β-sheet (denoted ‘β-sheet A’) to a thermodynamically-preferred 6-stranded conformation. The serpin fold (Fig. 1A) is highly conserved despite this functional diversity, with structural deviations largely restricted to extensions at the N- and C- termini and in a defined inter-helical region^1^. The unadorned core structure is epitomised by one of the most studied serpins, α_1_-antitrypsin (α_1_-AT).

Like most mesophillic serpins, α_1_-antitrypsin follows a three-state folding mechanism that proceeds from an unfolded state *via* an intermediate to a fully folded form^11^. The intermediate is of great interest, as it has been proposed to be the aggregating species central to the pathology of a number of hereditary conditions involving serpin deficiency^12,13^. Mutations, or environmental conditions, that increase the intermediate population by perturbing the energy landscape increase the formation of non-functional serpin polymers^12,14–16^. Conversely, minimal population of this intermediate has been observed in serpins produced by hyperthermophiles^17,18^, and is proposed as a mechanism whereby folding is possible, and activity is maintained, under destabilising conditions. The deficiency in functional α_1_-AT resulting from mutations such as Z, M_Malton_ or Siiyama is associated with neonatal hepatitis, liver disease, hepatocellular carcinoma and emphysema. Other serpins important to human physiology, such as antithrombin III, α_1_-antichymotrypsin, C1-inhibitor and plasminogen activator inhibitor-1, show a similar vulnerability to mutation^19^.

The formation of serpin polymers can be reduced under experimental conditions by stabilisation of the native state of the protein. This has been achieved by the rational introduction of point mutations to fill in surface cavities of α_1_-AT^20^, disulphide constraints of structural elements involved in conformational change^13,21–23^, the addition of osmolytes^23–25^ and random mutagenesis^26^. However, serpin function is intimately associated with a finely-tuned native state instability that is distributed throughout the molecule^23,27^, a context in which some, but not all, stabilising mutations compromise inhibitory activity^28^.

A further strategy that has been employed to increase the thermodynamic stability of a protein is the consensus approach to rational protein design. This technique identifies residues in a native protein that are putatively the most compatible with a given fold based on conservation between members of a protein family. Mutations have systematically been identified that are compatible with function and enhance stability for a range of proteins with include DNA binding proteins^29^, antibodies^30,31^, leucine rich repeat proteins^32^, and enzymes^33^. By extending this approach to the whole protein rather than selected residues, conserpin, an entirely artificial serpin, has recently been engineered^34^ with a sequence that reflects the most frequently observed residue at each site in an alignment of eukaroytic serpins^1^. This protein was found to adopt the canonical native, metastable conformation and showed a remarkable stability to inactivation by heat, an atypical fully reversible unfolding pathway, and an absence of polymer formation. Structural comparison with other eukaryotic serpins revealed features proposed to contribute to its stability, including stabilising interactions around α-helix D and the β-sheet B/C barrel, an extended electrostatic network in the ‘breach’ region, and tight packing in the hydrophobic core and at the β-sheet A/helix F interface. While conserpin showed a two-state unfolding profile atypical of serpins by intrinsic tryptophan fluorescence, the presence of folding intermediates was inferred from kinetic refolding experiments and 4,4′-dianilino-1,1′-binaphthyl-5,5′-disulfonic acid (bis-ANS) binding^34^.

Here we report the design, production and folding properties of a conserpin variant that incorporates the specificity-conferring reactive centre loop (RCL) residues of the disease-associated serpin, α_1_-AT. In common with its progenitor the resulting synthetic protein, cAT (consensus α_1_-AT), exhibited high thermal stability and reversible folding. We exploited the three tryptophans present in conserpin by generating single-tryptophan variants to probe unfolding in the proximity of helix F, the ‘breach’ at the top of β-sheet A, and helix H. These data, with the additional use of circular dichroism as a probe of change in secondary structure, extend the previous study by demonstrating recruitment of the whole molecule during folding. In combination with size-exclusion chromatography, we provide direct evidence of the presence of folding intermediate states inferred previously from kinetic data. We find the altered RCL favours inactivation by oligomerisation over transition to the latent form as observed for the parental protein, and utilise this property to assess the kinetic stability of the native state. Collectively, the data reflect a molecule with a highly efficient, concerted folding mechanism that minimises the accumulation of an aggregation-prone intermediate species as a result of a pronounced kinetic stability.

## Results

### Engineering a conserpin variant with α_1_-AT RCL residues

The design of conserpin has been described in detail previously^34^. The consensus sequence it embodies, when placed in a phylogenetic context^1^, has a slightly higher similarity to α_1_-AT-like, intracellular, and antithombin clades A-C over other subfamilies (Fig. 1B, *upper panel*). This is also reflected by a comparison between the conserpin structure^34^ and other serpins in the native conformation (Fig. 1B, *middle panel*) but not between loop-inserted conserpin and cleaved structures (Fig. 1B, *lower panel*). This highlights a dichotomy between the serpin metastable native conformation and the thermodynamically stabilised loop-inserted form.

The RCL sequence of conserpin was previously found to support inhibitory activity against trypsin, with a stoichiometry of inhibition (SI) of 1.8^34^, equivalent to a ~45% non-productive turnover rate. The conserpin sequence represents a hybrid of the subsite preferences of a broad spectrum of target proteases. However, as the interaction between two defined partners is generally determined by multiple specific subsite interactions within the binding cleft^35^, this consensus RCL may not fully capture the potential inhibitory activity of the conserpin scaffold against a single target. Furthermore, the conserpin RCL is 1–4 residues shorter on the P’ side than many inhibitory serpins. To introduce a sequence with a known cognate proteolytic profile, the P_7_ to P_2_′ region of the RCL was replaced by the corresponding region of α_1_-AT, to yield a functional variant, cAT. The purified protein (Fig. 1C) was found to exhibit a mixed α-helix/β-sheet far UV-circular dichroism profile consistent with a folded serpin (Fig. 1D).

### cAT is a functional serpin with α_1_-AT-like specificity

**Figure 2:**
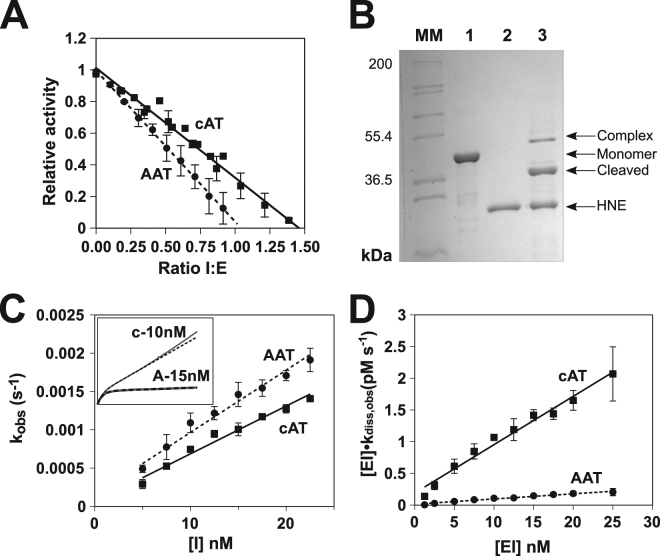
Serpin-enzyme complex formation by cAT. (**A**) Residual protease activity following the incubation at 25 °C of cAT with chymotrypsin at various molar ratios; the intercept of the linear regression with the abscissa is equivalent to the stoichiometry of inhibition for cAT (solid line) in comparison with α_1_-AT (dashed line). Error bars represent ± SEM for four independent experiments. (**B**) SDS-PAGE (10% w/v) of cAT incubated with human neutrophil elastase (HNE) at 37 °C for 2 hours. MM, molecular marker; lane 1, cAT control; lane 2, HNE; lane 4, cAT and HNE incubated at equimolar concentration. Monomer, cleaved and serpin-enzyme complex bands are labelled. (**C**) The inhibition of chymotrypsin by several concentrations of cAT and α_1_-AT was followed over time at 25 °C, from which the apparent second-order rate constant (k_obs_) was calculated. The slope of the relationship between inhibitor concentration and k_obs_ provides the uncorrected second-order rate constant, k_app_. Error bars represent ± SEM (n = 4). The inset graph shows representative progress curves of cAT (**c**) and α_1_-AT (**A**) with approximately equal k_obs_ values. (**D**) The dissociation of the complex between chymotrypsin and cAT or α_1_-AT was followed as the rate of regain of protease activity at 25 °C following dilution from 5 µM to the concentrations shown. The slope of the resulting regression provides the apparent rate constant, k_diss,app_. Error bars represent ± SEM (n = 4).

To determine whether the RCL substitution conferred cAT with an α_1_-AT-like activity, the SI was determined against chymotrypsin. SI is a measure of the efficiency of the inhibitory mechanism, governed by efficient loop incorporation and effective trapping of the tethered protease^36,37^. With a molar ratio of serpin-to-protease of 1.46 ± 0.03:1 (SEM, n = 4) to achieve full inhibition (Fig. 2A), this interaction was more efficient than the interaction of conserpin with trypsin, with around a third of cAT molecules following the substrate pathway. cAT was also found to form an SDS-stable inhibitory complex – a hallmark of many serpin-enzyme interactions^38^ – with human neutrophil elastase (Fig. 2B). This complex did not show evidence of secondary degradation by-products, in contrast to the interaction between conserpin and trypsin^34^. The second order association rate constant of cAT with chymotrypsin (k’_ass_), corrected for non-productive turnover, was 7.7 ± 0.2 × 10^5^ M^−1^ s^−1^ at 25 °C (SE of the regression, n = 4), slightly faster than the rate of interaction with α_1_-AT under the same experimental conditions, 6.9 ± 0.4 × 10^5^ M^−1^ s^−1^ (Fig. 2C). Thus cAT can present its α_1_-AT RCL in an effective orientation for engagement with a protease and is capable of a sufficiently rapid conformational change to act as an inhibitor.

The inhibitory progress curves did exhibit a difference in behaviour between the two proteins, however, with a noticeably higher residual protease activity for cAT that increased towards the end of the experiment (Fig. 2C, *inset*). This is characteristic of complex dissociation. To assess this, 5 µM inhibitory complex was diluted to 0–25 nM, and the regain of protease activity monitored by chromogenic substrate turnover. The apparent rate of complex dissociation observed for the cAT-chymotrypsin complex, 6.9 ± 1.0 × 10^−5^ s^−1^, was around 10-fold greater than that of the α_1_-AT-chymotrypsin complex, at 7.5 ± 1.2 × 10^−6^ s^−1^ (SEM, n = 4) (Fig. 2D). Coupled with a stoichiometry above parity, this is suggestive of a stability-function trade-off that yields an active but partially perturbed inhibitory mechanism.

### cAT forms higher-order species when heated

**Figure 3:**
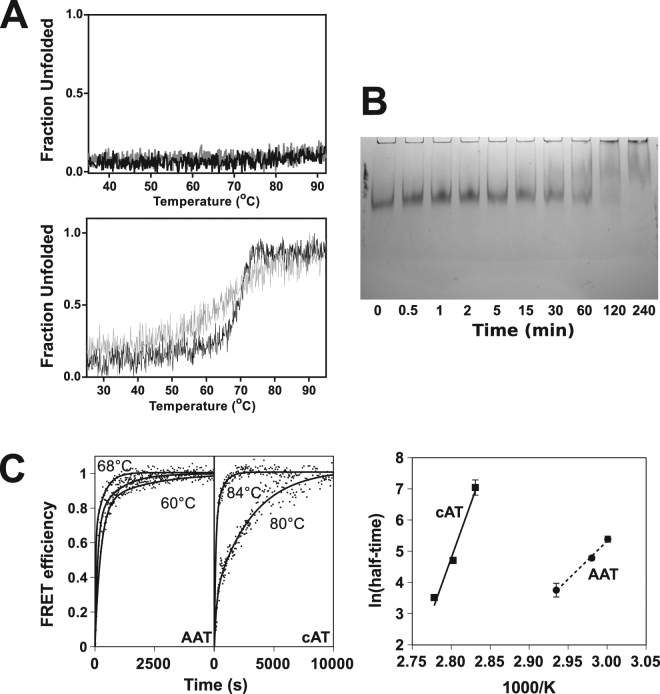
Heat-induced oligomerisation of cAT. (**A**) *Upper panel*: Thermal unfolding of 0.2 mg mL^−1^ cAT, heated from 25 °C to 95 °C at a rate of 1 °C min^−1^, monitored by the change in CD signal at 222 nm (black). The sample was then returned to the starting temperature at the same rate (grey). *Lower panel*: A representative thermal melt performed in the presence of 2 M of GdnHCl. (**B**) cAT (at 10 µM) was heated at 75 °C, 3 µL aliquots removed at various timepoints, and the oligomerisation state resolved by 6% non-denaturing PAGE. No monomer was visible at the conclusion of the experiment. (**C**) *Left panel*: cAT or α_1_-AT labelled with Atto 488 and Atto 594 fluorescent dyes at a concentration of 0.1 mg ml^−1^ in PBS was heated at temperatures between 80–87 °C and 60–68 °C, respectively, and the increase in FRET between the fluorophores determined over time. Points reflect representative data normalised between 0 and 1, with the curves of best fit as solid lines. *Right panel*: The natural logarithm half-times of the change in FRET were plotted against the reciprocal inverse absolute temperature; the slopes of the regressions are proportional to the apparent activation energy of polymerisation under the conditions of the experiment. Error bars reflect ± SEM (n = 4).

In common with the parental protein^34^, cAT was found to be resistant to heat-induced denaturation, with minimal detectable change in secondary structure over the course of a thermal denaturation assay from 25 °C to 95 °C at 1 °C min^−1^ (Fig. 3A, *upper*), requiring the addition of denaturant to unfold. In the presence of 2 M guanidine hydrochloride (GdnHCl) (Fig. 3A, *lower*), the unfolding temperature midpoint of denaturation (T_m_) of cAT was 70.0 ± 0.1 °C (SEM, n = 3), which is within 2.5 °C of the value for conserpin^34^. Following a decrease in temperature from 95 °C to 25 °C, the material showed no visible sign of precipitation, and retained ~50% activity (an SI value of 2.8 ± 0.2, SEM, n = 3) with respect to the pre-denaturation control. This partial loss of activity was not accompanied by a loss of circular dichroism (CD) signal, indicating that it was not associated with gross structural changes in, or significant precipitation of, the sample (Fig. 3A, *lower*). The thermal refolding curve revealed a similar midpoint transition of 69.5 °C albeit with a less co-operative behaviour than seen during unfolding, indicating that, in the presence of denaturant, the forward and reverse pathways of cAT are different.

Several serpins are known to form ordered long-chain polymers when heated due to population of an oligomerisation-prone intermediate state^23,39,40^. The latent conformation is an alternate monomeric end-state, in which the RCL is incorporated into β-sheet A without cleavage; this form can neither inhibit proteases nor polymerise^41^, and it also proposed to be accessed via an intermediate^42^. After heating at 76 °C for 5 hours, conserpin had been observed to fully convert to this monomeric latent conformation^34^. In contrast, incubation of cAT at 75 °C led to a gradual decrease in the monomer band intensity with a concurrent appearance of higher molecular mass species by non-denaturing PAGE, including a component unable to migrate into the gel (Fig. 3B). After four hours a complete loss of monomer was seen, with no residual monomer band that could indicate the presence of the latent species^41^. Thus the substitution of RCL residues in cAT confers behaviour more consistent with polymerisation-prone eukaryotic serpins, and by analogy the ability to adopt a polymerisation intermediate state.

To assess this possibility and follow polymerisation in real-time, mixtures of cAT and α_1_-AT (at 0.1 mg ml^−1^) labelled with Atto-488 and Atto-594 dyes were heated, and reaction progress monitored by a change in Förster resonance energy transfer (FRET). The resulting curves reported an increased proximity between donor and acceptor fluorophores to within ~80 Å, consistent with intermolecular association between differentially labelled proteins (Fig. 3C, *left*). The temperature dependence of the rate of polymerisation was then used to calculate the apparent activation energy of this oligomerisation (Fig. 3C, *right*). Under these conditions, the energetic barrier to polymerisation was found to be around 3-fold higher for cAT than α_1_-AT, at 139 ± 12 kcal mol^−1^ and 48 ± 6 kcal mol^−1^, respectively (±SE of regression, n = 4). Thus, the cAT native state exhibits significant kinetic stability against heat-induced inactivation.

### cAT unfolds via a poorly populated intermediate

**Figure 4:**
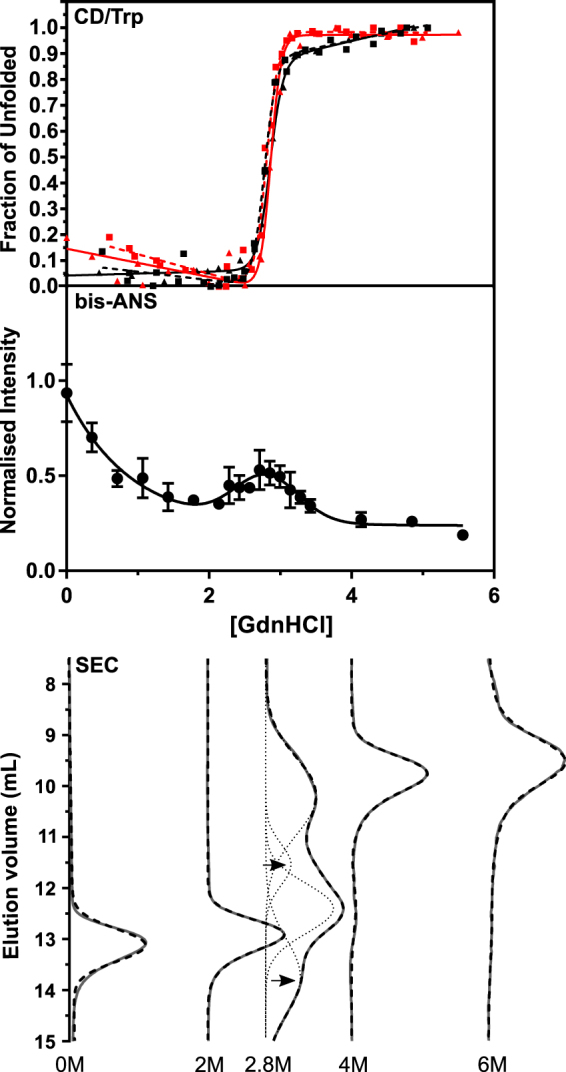
cAT populates an intermediate state over a narrow range of denaturant. *Upper panel*: Equilibrium GdnHCl-mediated unfolding (solid lines) and refolding (dotted lines) for cAT followed via the change in CD signal at 222 nm (black lines) and intrinsic fluorescence at 330 nm (red lines). Each dataset is the result of at least three independent experiments. A two-state unfolding curve was satisfactorily fit to the data; midpoints are listed in Table 1. *Middle panel*: cAT was incubated in varying concentrations of GdnHCl at 25 °C and bis-ANS added at 5 times the concentration of the protein. The fluorescence intensity was measured at 480 nm, with an excitation wavelength of 390 nm, and slit widths of 5 nm. Error bars reflect SD from three independent experiments, whose profiles were normalised according to their total integrated fluorescence intensity. The curve reflects the sum of an empirically-determined single exponential decay and Gaussian function. *Lower panel*: GdnHCl unfolding was monitored by size exclusion chromatography using a Superose 12 10/300 column, at the denaturant concentrations shown. The absorbance at 280 nm is shown in grey. The deconvoluted components of the 2.8 M sample are shown as dotted lines, the sums of the fitted components shown as dashed lines, and the experimental data as solid lines. Arrows indicate the expanded (top) and compact (bottom) intermediates.

In the seminal conserpin study^34^, intrinsic tryptophan fluorescence reported an apparently two-state equilibrium unfolding profile. This was inconsistent with data obtained using bis-ANS and rapid folding kinetics, which suggested the presence of a folding intermediate, and hence a three-state mechanism. This discrepancy could be explained either by a poorly populated intermediate, or well-populated intermediate with fluorescence properties very similar to either the native or unfolded state^34^. To investigate this further, GdnHCl-mediated equilibrium unfolding of cAT was undertaken whilst monitoring changes in far-UV CD signal, to use the global structure of the protein as an additional reporter of structural change. The resulting curves (Fig. 4, *upper panel*) showed an apparent two-state, strongly cooperative and near-contemporaneous transition in both fluorescence and CD, with the mid-point of denaturation (D_50%_) centred at 2.9 M. As noted for conserpin, this contrasts with the three-state unfolding profile exhibited by most serpins^12,40,43^. The equilibrium unfolding and refolding data were superimposable – consistent with a fully reversible folding pathway – and protein refolded from 6 M GdnHCl to 0.2 M GdnHCl had an inhibitory activity close to the starting value (SI of 1.7 ± 0.1; SEM, n = 3). Bis-ANS, an environment-sensitive probe whose quantum yield increases upon binding to hydrophobic regions of equilibrium intermediates^12,34,43^, was found to exhibit a small peak in mid-range concentrations of denaturant (Fig. 4, *middle panel*), centred at 2.8 ± 0.1 M GdnHCl (SE of global fit, n = 3). This corresponded well with fluorescence and far-UV CD D_50%_ values. Notably, the bis-ANS peak appeared over a noticeably more narrow concentration range than seen for α_1_-AT or α_1_-antichymotrypsin, with a width of ~1.7 M compared with 3 M for the latter two proteins^12,43^. These data are consistent with a minimally-populated folding intermediate and the simultaneous loss of secondary and tertiary structure at increasing concentrations of denaturant.

### Analytical gel filtration reveals species with expanded and compact conformations

Size exclusion chromatography (SEC) can be used to detect distinct intermediate ensembles with different hydrodynamic volumes to that of the native and unfolded conformations^18^. Accordingly, cAT was incubated in five different concentrations of GdnHCl chosen on the basis of the spectroscopic data, and the SEC elution profile of each sample determined (Fig. 4, *lower panel*). In the absence of denaturant, the native protein was found to elute at around 13 mL, while in 6 M GdnHCl, the expanded unfolded species eluted at 9.5 mL. At 2.8 M, which corresponds with the spectroscopic D_50%_ value and the peak in bis-ANS fluorescence, both of these species were evident, but with additional peaks at 11.5 mL and 13.7 mL. These peaks are indicative of a population of expanded molecules, as expected for the molten globule-like unfolding intermediate of α_1_-AT^44^, and additionally a compact ensemble with respect to the native state. Notably, re-folding rate data indirectly suggested that conserpin populates two distinct intermediates^34^, an inference which appears to be consistent with the species directly observed here. Interestingly, it has been proposed that the heat-induced intermediate of α_1_-AT shows compaction with respect to the native state^45,46^. At either 2 M or 4 M GdnHCl, these additional species were absent. In combination with the spectroscopic data, these profiles are consistent with the presence of a poorly populated intermediate ensemble, comprised of two species with distinct properties, which exist over a narrow denaturant concentration range.

### Single-tryptophan variants as probes of local folding

**Figure 5:**
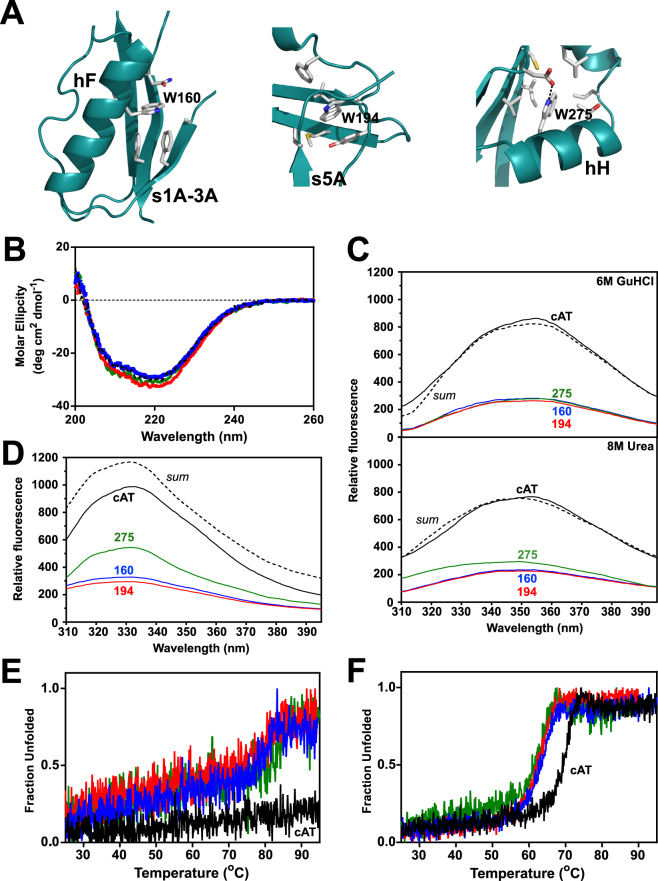
Properties of single-tryptophan mutants of cAT. (**A**) Residues and structural elements proximate to tryptophans 160, 194 and 275 (α_1_-AT numbering) present in the structure of native conserpin (5CDX) are shown. (**B**) Far-UV spectra of cAT_W160_ (blue) cAT_W194_ (red) and cAT_W275_ (green) are shown with respect to cAT (black dashes), at a concentration of 0.2 mg mL^−1^, using a path length of 0.1 cm, at 25 °C. (**C**) Fluorescence emission spectra for cAT (black), cAT_W160_ (blue) cAT_W194_ (red) and cAT_W275_ (green), in the presence of 6 M of GdnHCl (*upper panel*) and 8 M urea (*lower panel*). The dashed spectrum is the summation of all tryptophan mutants. Sample fluorescence, with excitation at 295 nm and 5 nm slit widths, was buffer-corrected. (**D**) As in panel *C*, but in the absence of denaturant. (**E**) Single-tryptophan variants cAT_W160_ (blue) cAT_W194_ (red) and cAT_W275_ (green) at 0.2 mg mL^−1^, heated from 25 °C to 95 °C at a rate of 1 °C min^−1^, with unfolding monitored by the change in CD signal at 222 nm. (**F**) As in panel *E*, performed in the presence of 2 M GdnHCl.

Tryptophan variants can be used as site-specific probes of the local folding behaviour of a protein, as described previously for α_1_-AT^47,48^ and plasminogen activator inhibitor-1^49^. The cAT sequence contains tryptophans at positions 160, 194 and 275 (all designations made using α_1_-AT numbering), corresponding with helix F, the loop connecting strand 3 of α-helix A and strand 4 of β-sheet C (situated in the ‘breach’ region at the top of β sheet A), and helix H respectively (Figs 1A and 5A). In order to use these residues as reporters of local structural change, single tryptophan variants were generated by systematically mutating two at a time to phenylalanine, to form the variants cAT_W160_, cAT_W194_, and cAT_W275_. The CD profiles of the resulting proteins were consistent with that of the wild-type cAT (Fig. 5B), and did not result in a loss of inhibitory activity (Table 1). Thus, the double tryptophan-to-phenylalanine substitutions did not substantially alter global structure or function.

**Table 1:** Comparison of cAT and single tryptophan mutants.

Variant	SI (pre-unfold)	SI (refold)	T_m_ (°C)	+GdnHCl T_m_ (°C)	D_50%_ (M)		∆*G*_D-N_ (kcal mol^−1^)
cAT	1.5 ± 0.1	1.7 ± 0.1	>95	69.9 ± 0.1	*Fl.CD*	2.9 (2.8)2.9 (2.8)	23 ± 3 19 ± 3
cAT_W160_	1.5 ± 0.2	1.4 ± 0.2	82.2 ± 0.3	64.0 ± 0.1	*Fl.CD*	2.5 (2.6)2.6 (2.6)	13 ± 3 13 ± 2
cAT_W194_	1.2 ± 0.1	1.1 ± 0.1	83.1 ± 0.3	64.1 ± 0.1	*Fl.CD*	2.5 (2.6)2.5 (2.5)	18 ± 3 13 ± 2
cAT_W275_	1.5 ± 0.1	1.6 ± 0.1	77.6 ± 0.1	61.9 ± 0.1	*Fl.CD*	2.7 (2.7)2.6 (2.6)	15 ± 2 11 ± 1

The SI was determined against chymotrypsin prior to unfolding in 6 M GdnHCl and after refolding by dilution back to a final GdnHCl concentration of 0.2 M. SI errors reflect ± SE from the fit to the data (n = 2 or 3). Thermal unfolding was monitored by the change in CD signal, in the absence and presence of 2 M GdnHCl; T_m_ errors are SEM from three independent experiments. Refold D_50%_, shown in parentheses, was derived by unfolding protein in 6 M GdnHCl and then diluting it back into 0.2 M GdnHCl; midpoint and ∆*G*_D-N_ values were calculated from equilibrium unfolding intrinsic fluorescence and CD profiles, as indicated.

In the presence of 6 M GdnHCl, the intrinsic fluorescence emission spectra of the denatured proteins were fully additive (Fig. 5C, *upper panel*). Spectra of cAT and variants were also recorded in the absence of denaturant (Fig. 5D). The sum of the emission spectra of all single tryptophan variants yielded a maximal intensity about 120% of that of cAT, with comparable peak emission wavelengths at 331–332 nm. A previous analysis of plasminogen activator inhibitor-1, with three of the four tryptophan residues at identical positions to those in cAT, revealed significant quenching of Trp275 by Trp194^49^. In the context of similar inhibitory activity, far-UV CD profiles and the peak fluorescence emission wavelength across the three cAT variants, this 20% increase in intensity was therefore more likely the consequence of resonance energy transfer in cAT rather than the result of marked structural perturbation.

Intrinsic fluorescent scans were also performed in the presence of 8 M urea. Two of the variants had identical emission profiles to that seen when unfolded in 6 M GdnHCl. The exception, cAT_W275_ (Fig. 5C, *lower panel*), displayed an intensity at the emission maximum slightly higher than the other variants coupled with a −5 nm blue shift. This indicates that full solvation was not achieved at position Trp275 and some structure persists around helix H. The vicinity of helix H is thus more resistant to unfolding, as seen previously in α_1_-antichymotrypsin^50^; the folding nucleus around which the rest of the serpin scaffold condenses therefore appears to be conserved in cAT.

### Tryptophan residues contribute to the stability of cAT

Thermal unfolding experiments were used to assess whether the double tryptophan substitutions resulted in a change in stability. In the absence of denaturant, the variants exhibited an observable unfolding profile, with T_m_ values of approximately 80 °C (Table 1 and Fig. 5E). This is appreciably lower than wild-type cAT but considerably higher than any single eukaryotic serpin studied. Similarly, in the presence of 2 M GdnHCl, the variants had a T_m_ of 6–8 °C lower than parental cAT (see Fig. 5F and Table 1). These data highlight the importance of the hydrophobic packing mediated by each of these residues, within the key helix F/β-sheet A, breach and sheet B/C barrel regions (Fig. 5A).

### cAT folding and unfolding is cooperative

**Figure 6:**
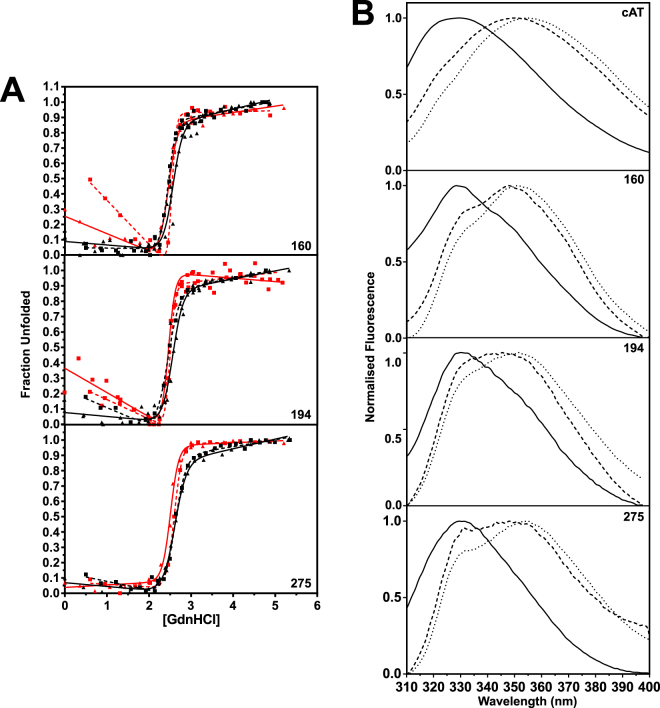
Equilibrium unfolding of single-tryptophan variants of cAT. (**A**) Equilibrium GdnHCl-mediated unfolding (solid lines) and refolding (dotted lines) for cAT_W160_, cAT_W194_ and cAT_W275_ followed by the change in CD signal at 222 nm (black lines) and intrinsic fluorescence at 330 nm (red lines). Each dataset is the result of at least three independent experiments. A two-state unfolding curve was fitted to the data. The concentration at which half the protein is unfolded (D_50%_) or refolded (D_50% refold_) is shown in Table 1. (B) Normalised fluorescence emission spectra of parental cAT, cAT_W160_, cAT_W194_ and cAT_W275_ in buffer without denaturant (—), and in the presence of 2.6 M or 3 M of GdnHCl (---) and 6 M GdnHCl (···). All scans were conducted at 25 °C, with an excitation wavelength of 295 nm and slit widths of 5 nm.

Equilibrium unfolding experiments were then performed on the mutants, with measurements by both CD and intrinsic fluorescence (Fig. 6A). The calculated D_50%_ values (Table 1) reflected a marginally lower thermodynamic stability than the wild type cAT protein (2.5–2.6 M as compared with 2.9 M) with a ∆∆*G*_D-N_ of greater than 4.4 kcal mol^−1^ between cAT and the single tryptophan variants. These data point to the importance of the local interactions made by the tryptophan residues. However, reversibility was not affected: similar curves were seen when the refolding of each variant was monitored in the same manner. The close correspondence in behaviour reported by tryptophan residues on different structural elements, by global (CD) and by local (intrinsic fluorescence) measures of unfolding, highlight the highly cooperative nature of cAT folding.

### Intrinsic fluorescence spectra reveal the presence of a folding intermediate

Fluorescence scans of α_1_-AT at the midpoint of chemical denaturation exhibited a red shift from 330 nm to 343 nm, which has been ascribed to the presence of an intermediate ensemble^47^. Similar scans were conducted here at 3 M and 2.6 M GdnHCl for cAT and the tryptophan variants, respectively (Fig. 6B). Under these conditions, all double mutants showed a λ_max_ of approximately 348 nm, signifying the partial exposure of each individual tryptophan to the solvent due to local conformational changes. This common behaviour is consistent with a molten globule intermediate^51^. These data, in combination with the results obtained using bis-ANS and SEC, support the conclusion that cAT folds through a three-state mechanism via a molten globule intermediate. Therefore, the reversible nature of cAT unfolding is a consequence of a poorly populated, rather than absent, folding intermediate, facilitated by a native state with pronounced thermodynamic and kinetic stability and a highly efficient and cooperative folding pathway.

## Discussion

Serpins are present in every taxonomic phylum, including prokaryotes that live at extremes of temperature. However, well-characterised serpins relevant to human physiology generally possess a finely-balanced stability easily perturbed by mutation, which on the face of it appears to be a necessary compromise to maintain the unique mechanism of action. It follows that prokaryotic hyperstable serpins should therefore be the product of specialised evolutionary adaptations to an intrinsically unstable scaffold. It is notable then that conserpin, a protein representative of eukaryotic serpins, exhibits both hyperstability and inhibitory activity. Indeed, this protein exhibits structural features that are distinct from the kinds of changes typically associated with adaptation to destabilising environments. Instead, it is proposed that the folding landscape has been smoothed, reducing opportunities for populating aggregation-prone intermediate states^34^.

The whole-protein consensus approach to the design of conserpin was simple: each residue corresponds to the most frequently observed amino acid at each position in an alignment of eukaryotic serpins. The underlying premise is that, over the course of evolution, extensive residue-level sampling has occurred, and changes which are the least incompatible with function and stability will be over-represented in modern-day sequences^52^. This is an approach distinct from the derivation of an ancestral sequence, which reconstructs a protein based on phylogenetic relationships, and which by definition attempts to negate the influence of changes in sequence that have occurred subsequent to the chosen branch-point. Thus it is unsurprising that there is a higher representation of residues in conserpin from the most populous phylogenetic clades, α_1_-AT-like clade A and intracellular clade B (Fig. 1B).

While the consensus requirements of the N-terminal RCL hinge region are well-known^53^, the specificity of a serpin is reliant on an effective substrate-like interaction between the C-terminal region of the RCL and a target protease. This in turn depends on protease subsite preferences and active site topology^54^, in which both RCL sequence and length play a role. Thus the observation that around 45% of conserpin molecules are cleaved non-productively may be a consequence of an RCL sequence built on consensus principles. To test this, and generate a tool protein with a known inhibitory profile, the specificity-determining residues of the α_1_-AT RCL were introduced onto the conserpin scaffold. Whilst exhibiting a comparable rate of association for α_1_-AT with chymotrypsin, and a higher degree of inhibition with respect to conserpin, this change did not convert the resulting protein into a stoichiometric inhibitor (Fig. 2A and C). Additionally, the inhibitory complex exhibited compromised stability with respect to α_1_-AT, with a gradual regain of protease activity at an accelerated rate (Fig. 2D). The basis for this is not immediately clear; the two have a comparable RCL length and the resting place of the inhibited protease is structurally similar in both. It has been shown that the protease catalytic triad can be distorted to various extents in some complexes^55^ and that partial translocation of a protease results in a relatively unstable inhibitory state^56^. It is possible that the instability is due to an impedance of full translocation, or an otherwise incompletely inhibited catalytic triad. Thus, it appears that the less efficient inhibitory mechanism is a trade-off for the gain in stability.

Like its progenitor protein, cAT exhibited a remarkable thermal stability at odds with the eukaryotic serpins from which the consensus sequence was derived (Fig. 3A); the only naturally-occurring serpin characterised to date with a higher stability is aeropin, produced by an archaeon that lives at temperatures close to 100 °C^18^. This was associated with a pronounced increase in the kinetic barrier to polymerisation (Fig. 3C), while thermodynamic stability with respect to the unfolded state was partially attributable to the three tryptophan residues in helix F, at the top of β-sheet A, and helix H (Fig. 5E). The lack of distinction between denaturant and thermal measures of stability (Figs 5E,F and 6A) reinforces a contribution to global stability and the high degree of cooperativity between the three locales during folding. It has been reported that introduction of a tryptophan at position 160 of α_1_-AT elevated the T_m_ from 59 to 65 °C^48^; correspondingly, this side-chain contributes to an enhanced packing with β-sheet A in the conserpin structure^34^. Trp194 is the most highly conserved of the three, situated in the breach region, and substitution with a phenylalanine residue has been found to decrease the kinetic stability of α_1_-antichymotrypsin^57^, although not the thermodynamic stability of α_1_-AT^47^. The loss of Trp275 does not compromise reversibility or lead to a disproportionate impact on thermodynamic stability with respect to the other tryptophan residues; thus, it does not appear to be required for the putative folding nucleus (Fig. 5C, *lower panel*).

The equilibrium unfolding and refolding profiles of cAT and its tryptophan variants were consistent with those observed for conserpin: they exhibited a two-state transition, showed full reversibility, and exhibited a high degree of cooperativity (Figs 4 and 6A). In contrast, all characterised eukaryotic serpins have been found to unfold via an intermediate species^18^ often described as molten globule-like in structure^51^, and no other eukaryotic serpin has been shown to unfold reversibly and refold fully. Accordingly, intrinsic fluorescence spectra of individual tryptophan residues and analytical size exclusion chromatography confirmed the presence of a distinct expanded intermediate ensemble (Figs 4 and 6B). The resolution of two novel components over a narrow denaturant range is consistent with indirect evidence of intermediates inferred from rapid folding of conserpin^34^ and with the observation of distinct transition midpoints in plasma α_1_-AT reported by different spectroscopic approaches^58^. The concurrent appearance of these intermediates at equilibrium under restrictive denaturant concentrations suggests that they are interconvertible, directly or indirectly. In the context of folding from a denatured state, this further supports a highly efficient collapse from expanded to compact ensembles.

Polymers and the inactive latent conformation form via intermediate states that can be induced under destabilising conditions^23,39,40,42^. In α_1_-AT, the degree of native state perturbation is associated with generation of two different polymer configurations, attained through two different intermediates - a ‘denaturant-induced’ expanded molten globule unfolding intermediate^44^ and a compact ‘heat-induced’ polymerisation intermediate^45,46^. Whilst definitive equivalence cannot be determined from these data, it remains noteworthy that the hydrodynamic volumes of cAT intermediate species are consistent with those implicated in α_1_-AT polymerisation. Further investigation would be required to establish whether the intermediate states identified here represent branch points leading to different conformational outcomes. Nevertheless, with intermediates apparently representing an obligate component of the serpin folding pathway, it is most likely their low abundance that asserts the greatest influence on thermal stability and reversibility of unfolding of cAT. Indeed, our data combined with published observations of conserpin^34^ and aeropin^18^ support a general mechanism in which serpin stability against misfolding and polymerisation is achieved not by elimination of such intermediates, but by limiting the extent to which they are populated.

When considering mechanisms of inactivation, a noteworthy divergence in behaviour of cAT from conserpin is the appearance of higher-order species during prolonged heating at high temperature (Fig. 3B). Under these conditions the parental conserpin protein also undergoes an inactivating change, but in contrast does so in a unimolecular fashion, resulting in formation of the latent species^34^. The prevailing evidence is that polymerisation and latency arise through a common process: both have been observed to occur simultaneously with neuroserpin^42^, Z α_1_-AT and M α_1_-AT in the presence of citrate^41,59^, and thus have been proposed to represent alternate outcomes determined by a decision point along the pathway^42^. Structurally, both forms require a mobile strand 1 of β-sheet C, and at least partial insertion of the RCL as an additional central 6^th^ strand in β-sheet A^14,21,60,61^. As cAT and conserpin undergo conformational change with a similar half-time of around 1-2 hours under equivalent conditions, the substitution of the α_1_-AT RCL residues has not made cAT substantially more prone to inactivation, but instead shifted the balance towards the formation of oligomers. A basis for this could be greater compatibility of the P_7_-P_1_′ RCL residues with the inserted state, in line with an improved stoichiometry of inhibition relative to the parental conserpin protein (Fig. 2A). Whether this is the case or not, these data indicate that an attempt to further limit inactivating conformational change should focus in the first instance on residues outside of the RCL.

In α_1_-AT, the intermediate ensemble has been characterised as partially folded, possessing an intact β-sheet B and helices G and H, an expanded β-sheet A and a disrupted helix F^47,48,51,62,63^. This is compatible with the presence of residual structure in the vicinity of Trp275 (on helix H) in 8 M urea and the disordered breach and helix F regions (Trp160 and Trp194 respectively) observed here. By extension, this suggests that not only is the native structure and function of the consensus serpin protein conserved, but the folding pathway is as well.

The development of a synthetic serpin using a consensus strategy was found to yield a functional protein that is highly thermostable, resistant to polymerisation and able to fold reversibly. This study has provided direct evidence for two transiently populated intermediates, and the use of single-tryptophan variants has revealed the folding pathway to exhibit a high degree of cooperativity and conservation of the folding nucleus. Substitution of the RCL has improved inhibitory efficiency but suggests that that further optimisation of the conserpin core may be necessary to maximise this. Even so, with a fully reversible folding pathway, pronounced stability, and adaptable specificity, conserpin and the derivatives characterised here provide a useful platform for the investigation of conformational change and stability in the serpin superfamily.

## Methods

### Materials and software

All reagents were from Sigma Aldrich unless specified. The concentration of stock solutions of guanidine hydrochloride (GdnHCl) was determined using refractive index measurements^64^. Molecular weight markers were from Life Technologies and Fermentas. A primary mouse anti-histidine tag antibody (AbD Serotec) and secondary sheep anti-mouse antibody (Chemicon) were used for western blots. Bovine α-chymotrypsin was stored in 1 mM HCl. Human neutrophil elastase (HNE) was from Calbiochem and prepared in 50 mM sodium acetate, 200 mM NaCl, pH 5. Non-linear regression and numerical calculation of reaction half-times was performed using Prism (GraphPad Inc.) and GNU Octave. Structural representations were generated using Pymol (Schrodinger Inc.).

### Design of the consensus serpin (cAT)

The engineering of the conserpin sequence has been described previously^34^. Residues P_7_-P_1_′ (GVEIVPRS) were replaced with the P_7_-P_2_′ region of the α_1_-AT RCL (FLEAIPMSI). The constructs used for the single tryptophan mutation experiments were codon-optimised for *E. coli* and commercially synthesised by DNA 2.0 (USA).

### Expression constructs

Plasmids encoding cAT and the mutants cAT_W160_ (W194F/W275F), cAT_W194_ (W160F/W275F), and cAT_W275_ (W160F/W194F) were generated using ligation-independent cloning with the pLIC-HIS vector using standard protocols; these constructs were transformed into BL21(DE3) *E. coli* and subjected to small scale expression^65^. Colonies were screened for expression using a crude activity assay in which 90 µL of the lysate was mixed with 10 µL of 10 µM of chymotrypsin.

### Protein expression and purification

Protein was expressed using Overnight Express™ Instant TB Medium (Merck-Millipore) as described previously^65^. Cells were harvested, lysed in 40 mL lysis buffer (10 mM imidazole, 25 mM NaH_2_PO4, 300 mM NaCl, pH 8.0) supplemented with 2 mM β-mercaptoethanol, 0.125 mM PMSF, 0.25 mg mL^−1^ lysozyme and 1 mg of DNAse I. Following centrifugation, the soluble fraction was filtered through a 0.22 µm filter membrane (Millipore) and applied to a pre-equilibrated 5 ml HisTrap HP column (GE Healthcare), washed with 6 volumes of 20 mM imidazole, 25 mM NaH_2_PO4, 300 mM NaCl, pH 8.0 and eluted with 500 mM imidazole, 25 mM NaH_2_PO4, 300 mM NaCl, pH 8.0. Peak fractions were loaded onto a Superdex 200 16/60 column and eluted with 50 mM Tris, 90 mM NaCl, pH 8.0; protein was concentrated to ~300 µM and stored at −80 °C.

### Characterisation of inhibitory properties

The SI and association rate constant (k_ass_) of cAT and single tryptophan variants against bovine chymotrypsin was determined as described previously^66^ using protease assay buffer (20 mM Tris, 100 mM NaCl, 0.1% (w/v) PEG 8000, 10 mM CaCl_2_, pH8.0) and 200 µM N-succinyl-Ala-Ala-Pro-Phe-p-nitroanilide substrate. The refolding SI was measured using protein unfolded in 6 M of GdnHCl, 90 mM NaCl and 50 mM Tris, pH8.0 for 2 hours, and refolded into 50 mM Tris, 90 mM NaCl, pH 8.0, so the final concentration of the GdnHCl was 0.2 M, for an additional 2 hours. For complex dissociation experiments, 5 µM serpin-chymotrypsin complex was diluted 200–2000-fold into assay buffer containing substrate, and the resulting progress curves describing the regain of activity were fit by a quadratic equation as described^67^ with the turnover number calculated separately for each discrete experiment.

### Heat-induced oligomerisation

The disappearance of monomer was analysed by incubating 10 µL of 10 µM of cAT protein samples at 75 °C for different times. 3 µL of the protein sample was then added to 1 µL of native-PAGE loading buffer and resolved by 6% native-PAGE as described^68^. For FRET experiments, α_1_-AT or cAT, in phosphate-buffered saline (PBS), were incubated with a 2–3-fold molar excess of either NHS-Atto 488 or NHS-Atto 594 dyes (Atto-Tec, Germany) in separate reactions for 4 hours at 25 °C. Following the quenching of the reaction by 10 mM hydroxylamine, the preparations were were separated from unconjugated dye by anion exchange chromatography as described^66^. Polymerisation experiments, with protein at a concentration of 0.1 mg ml^−1^ in PBS and a total volume of 20 µl, were performed using a Mastercycler Realplex4 instrument (Eppendorph) and the resulting curves were well-described by a double exponential equation as noted previously^66^. The temperature dependence of these reaction half-times was assessed using an Arrhenius plot, with the apparent activation energy (E_act,app_) calculated by multiplying the slope of the regression by the gas constant.

### Circular dichroism scans and thermal denaturation

Circular dichroism (CD) measurements were performed on a Jasco J-815 CD spectrometer (Jasco) at a protein concentration of 0.2 mg mL^−1^ with 90 mM NaCl and 50 mM Tris, pH 8.0 using a quartz cell with a path-length of 0.1 cm. Far-UV scans were performed between 200–260 nm for samples in 90 mM NaCl and 50 mM Tris, pH 8.0. For thermal denaturation, a heating rate of 1 °C min^−1^ from 25 °C to 95 °C was used, with the change in signal measured at 222 nm. Refolding was measured directly after the thermal melt by holding the temperature at 95 °C for 1 min before the temperature was decreased to 25 °C at the same rate. The midpoint of transition (T_m_) was obtained by fitting the data with a Boltzmann sigmoidal curve in accordance with the method described^69^ for both forward and reverse thermal denaturation experiments.

### Size exclusion chromatography (SEC)

SEC was performed on a Superose 12 10/300 gel filtration column equilibrated with the respective buffer that the protein was incubated in: 0 M, 2 M, 2.8 M, 4 M or 6 M GdnHCl in 90 mM NaCl and 50 mM Tris, pH8.0. The elution profiles, reflecting the absorbance at 280 nm, were fit to the sum of one or more Gaussian functions.

### Tryptophan fluorescence scans

Intrinsic fluorescence was measured using a FluoroMax-4 spectrofluorometer (HORIBA Jobin Yvon) with 0.5 µM of each protein in a 1 cm path-length quartz cell at 25 °C. The excitation wavelength (λ_ex_) was 295 nm and the emission wavelength (λ_em_) was 330 nm, with 5 nm slit widths.

### Equilibrium unfolding and refolding

Equilibrium unfolding was performed as described^18^ using both CD and intrinsic fluorescence. Refolding curves were obtained through first unfolding the protein in 6 M of GdnHCl, 90 mM NaCl and 50 mM Tris, pH8.0 for 2 hours and then refolding back into buffer containing various concentration of GdnHCl with 50 mM Tris, 90 mM NaCl, pH 8.0 for an additional 2 hours. The fraction of unfolded was plotted against the final GdnHCl concentration.

### Sequence and structural comparisons

MEGA 6^70^ was used to calculate the average pairwise sequence identity between conserpin and aligned eukaryotic serpins separated into phylogenetic clades^1^. To produce sub-alignments in which the most variable sites were removed at different thresholds, positions were ranked by their Kabat variability score (calculated as the number of amino acids at a site ÷ the frequency of the most common amino acid). For structural comparisons, the native (PDB 5CDX) and latent (PDB 5CDZ) conformations of conserpin^34^ were aligned to native and loop-inserted serpin structures, respectively, using SUPERPOSE^71^. Two rounds of superpositions were performed: following the first, those residue positions in conserpin that failed to match in any pairwise comparison were excluded the subsequent round, following which root-mean square deviations (RMSD) were calculated.

## Electronic supplementary material

Supplementary Information

## References

[1] Irving JA, Pike RN, Lesk AM, Whisstock JC (2000). Phylogeny of the serpin superfamily: implications of patterns of amino acid conservation for structure and function. Genome Res.

[2] Irving JA (2002). Serpins in prokaryotes. Mol Biol Evol.

[3] Ragg H, Lokot T, Kamp PB, Atchley WR, Dress A (2001). Vertebrate serpins: construction of a conflict-free phylogeny by combining exon-intron and diagnostic site analyses. Mol Biol Evol.

[4] Ray CA (1992). Viral inhibition of inflammation: cowpox virus encodes an inhibitor of the interleukin-1 beta converting enzyme. Cell.

[5] Schick C (1998). Cross-class inhibition of the cysteine proteases cathepsins K, L, and S by the serpin squamous cell carcinoma antigen 1: a kinetic analysis. Biochemistry.

[6] Irving JA (2002). Evidence that serpin architecture intrinsically supports papain-like cysteine protease inhibition: engineering alpha(1)-antitrypsin to inhibit cathepsin proteases. Biochemistry.

[7] Dafforn TR, Della M, Miller AD (2001). The molecular interactions of heat shock protein 47 (hsp 47) and their implications for collagen biosynthesis. J Biol Chem.

[8] Zou Z (1994). Maspin, a serpin with tumor-suppressing activity in human mammary epithelial cells. Science.

[9] Anfinsen CB (1973). Principles that Govern the Folding of Protein Chains. Science.

[10] Seo EJ, Im H, Maeng JS, Kim KE, Yu MH (2000). Distribution of the native strain in human alpha 1-antitrypsin and its association with protease inhibitor function. J Biol Chem.

[11] Powell LM, Pain RH (1992). Effects of glycosylation on the folding and stability of human, recombinant and cleaved a1-antitrypsin. J Mol Biol.

[12] Knaupp AS, Levina V, Robertson AL, Pearce MC, Bottomley SP (2010). Kinetic instability of the serpin Z alpha1-antitrypsin promotes aggregation. J Mol Biol.

[13] Yamasaki M, Li W, Johnson DJ, Huntington JA (2008). Crystal structure of a stable dimer reveals the molecular basis of serpin polymerization. Nature.

[14] Lomas DA, Evans DL, Finch JT, Carrell RW (1992). The mechanism of Z alpha 1-antitrypsin accumulation in the liver. Nature.

[15] Mast AE, Enghild JJ, Salvesen G (1992). Conformation of the reactive site loop of alpha 1-proteinase inhibitor probed by limited proteolysis. Biochemistry.

[16] Knaupp AS, Bottomley SP (2011). Structural change in beta-sheet A of Z alpha(1)-antitrypsin is responsible for accelerated polymerization and disease. J Mol Biol.

[17] Zhang Q (2007). The N terminus of the serpin, tengpin, functions to trap the metastable native state. EMBO Rep.

[18] Cabrita LD, Irving JA, Pearce MC, Whisstock JC, Bottomley SP (2007). Aeropin from the extremophile Pyrobaculum aerophilum bypasses the serpin misfolding trap. J Biol Chem.

[19] Lomas DA, Mahadeva R (2002). Alpha-1-Antitrypsin polymerization and the serpinopathies: pathobiology and prospects for therapy. Journal of Clinical Investigation.

[20] Gooptu B (2009). Crystallographic and cellular characterisation of two mechanisms stabilising the native fold of alpha1-antitrypsin: implications for disease and drug design. J Mol Biol.

[21] Chang WS (1997). Importance of the release of strand 1C to the polymerization mechanism of inhibitory serpins. Protein Sci.

[22] Baek JH (2007). Probing the local conformational change of alpha1-antitrypsin. Protein Sci.

[23] Irving JA, Haq I, Dickens JA, Faull SV, Lomas DA (2014). Altered native stability is the dominant basis for susceptibility of alpha1-antitrypsin mutants to polymerization. Biochem J.

[24] Devlin GL, Parfrey H, Tew DJ, Lomas DA, Bottomley SP (2001). Prevention of polymerization of M and Z alpha1-Antitrypsin (alpha1-AT) with trimethylamine N-oxide. Implications for the treatment of alpha1-at deficiency. Am J Respir Cell Mol Biol.

[25] Sharp LK (2006). Sugar and alcohol molecules provide a therapeutic strategy for the serpinopathies that cause dementia and cirrhosis. FEBS J.

[26] Lee KN, Im H, Kang SW, Yu M-H (1998). Characterization of a human a1-antitrypsin variant that is as stable as ovalbumin. J Biol Chem.

[27] Seo EJ, Lee C, Yu MH (2002). Concerted regulation of inhibitory activity of alpha 1-antitrypsin by the native strain distributed throughout the molecule. J Biol Chem.

[28] Im H, Seo EJ, Yu M-H (1999). Metastability in the inhibitory mechanism of human a1-antitrypsin. J Biol Chem.

[29] Nikolova PV, Henckel J, Lane DP, Fersht AR (1998). Semirational design of active tumor suppressor p53 DNA binding domain with enhanced stability. Proc Natl Acad Sci USA.

[30] Steipe B, Schiller B, Pluckthun A, Steinbacher S (1994). Sequence statistics reliably predict stabilizing mutations in a protein domain. J Mol Biol.

[31] Knappik A (2000). Fully synthetic human combinatorial antibody libraries (HuCAL) based on modular consensus frameworks and CDRs randomized with trinucleotides. J Mol Biol.

[32] Stumpp MT, Forrer P, Binz HK, Pluckthun A (2003). Designing repeat proteins: modular leucine-rich repeat protein libraries based on the mammalian ribonuclease inhibitor family. J Mol Biol.

[33] Anbar M, Gul O, Lamed R, Sezerman UO, Bayer EA (2012). Improved thermostability of Clostridium thermocellum endoglucanase Cel8A by using consensus-guided mutagenesis. Appl Environ Microbiol.

[34] Porebski BT (2016). Smoothing a rugged protein folding landscape by sequence-based redesign. Sci Rep.

[35] Schecter I, Berger A (1967). On the size of the active site in proteases. I. Papain. Biochem Biophys Res Commun.

[36] Zhou A, Carrell RW, Huntington JA (2001). The serpin inhibitory mechanism is critically dependent on the length of the reactive center loop. J Biol Chem.

[37] Huntington JA, Read RJ, Carrell RW (2000). Structure of a serpin-protease complex shows inhibition by deformation. Nature.

[38] Lawrence DA (1995). Serpin-Protease complexes are trapped as stable acyl-enzyme intermediates. Journal of Biological Chemistry.

[39] Dafforn TR, Mahadeva R, Elliott PR, Sivasothy P, Lomas DA (1999). A kinetic mechanism for the polymerization of alpha1-antitrypsin. J Biol Chem.

[40] James EL, Bottomley SP (1998). The mechanism of a1-antitrypsin polymerization probed by fluorescence spectroscopy. Arch Biochem Biophys.

[41] Lomas DA, Elliott PR, Chang WS, Wardell MR, Carrell RW (1995). Preparation and characterization of latent alpha 1-antitrypsin. J Biol Chem.

[42] Chiou A (2009). Probing neuroserpin polymerization and interaction with amyloid-beta peptides using single molecule fluorescence. Biophys J.

[43] Pearce MC, Rubin H, Bottomley SP (2000). Conformational change and intermediates in the unfolding of alpha 1-antichymotrypsin. J Biol Chem.

[44] Tsutsui Y, Dela Cruz R, Wintrode PL (2012). Folding mechanism of the metastable serpin alpha1-antitrypsin. Proc Natl Acad Sci USA.

[45] Tsutsui Y, Kuri B, Sengupta T, Wintrode PL (2008). The structural basis of serpin polymerization studied by hydrogen/deuterium exchange and mass spectrometry. J Biol Chem.

[46] Ekeowa UI (2010). Defining the mechanism of polymerization in the serpinopathies. Proc Natl Acad Sci USA.

[47] Tew DJ, Bottomley SP (2001). Probing the equilibrium denaturation of the serpin alpha(1)-antitrypsin with single tryptophan mutants; evidence for structure in the urea unfolded state. J Mol Biol.

[48] Cabrita LD, Whisstock JC, Bottomley SP (2002). Probing the role of the F-helix in serpin stability through a single tryptophan substitution. Biochemistry.

[49] Verheyden S, Sillen A, Gils A, Declerck PJ, Engelborghs Y (2003). Tryptophan properties in fluorescence and functional stability of plasminogen activator inhibitor 1. Biophys J.

[50] Pearce MC, Cabrita LD, Rubin H, Gore MG, Bottomley SP (2004). Identification of residual structure within denatured antichymotrypsin: implications for serpin folding and misfolding. Biochem Biophys Res Commun.

[51] Tsutsui Y, Wintrode PL (2007). Cooperative unfolding of a metastable serpin to a molten globule suggests a link between functional and folding energy landscapes. J Mol Biol.

[52] Porebski BT, Buckle AM (2016). Consensus protein design. Protein Eng Des Sel.

[53] Hopkins PC, Carrell RW, Stone SR (1993). Effects of mutations in the hinge region of serpins. Biochemistry.

[54] Ye S (2001). The structure of a Michaelis serpin-protease complex. Nat Struct Biol.

[55] Plotnick MI (2002). Heterogeneity in serpin-protease complexes as demonstrated by differences in the mechanism of complex breakdown. Biochemistry.

[56] Liu L, Mushero N, Hedstrom L, Gershenson A (2007). Short-lived protease serpin complexes: partial disruption of the rat trypsin active site. Protein Sci.

[57] Pearce MC, Cabrita LD, Ellisdon AM, Bottomley SP (2007). The loss of tryptophan 194 in antichymotrypsin lowers the kinetic barrier to misfolding. FEBS J.

[58] Herve M, Ghelis C (1990). Conformational changes in intact and papain-modified alpha 1-proteinase inhibitor induced by guanidinium chloride. Eur J Biochem.

[59] Tan L (2015). Characterising the association of latency with alpha(1)-antitrypsin polymerisation using a novel monoclonal antibody. Int J Biochem Cell Biol.

[60] Yamasaki, M., Sendall, T. J., Pearce, M. C., Whisstock, J. C. & Huntington, J. A. Molecular basis of alpha1-antitrypsin deficiency revealed by the structure of a domain-swapped trimer. *EMBO Rep* **12**, 1011-1017, embor2011171 [pii] 10.1038/embor.2011.171 (2011). 10.1038/embor.2011.171 PMC3185345 21909074

[61] Mottonen J (1992). Structural basis of latency in plasminogen activator inhibitor-1. Nature.

[62] James EL, Whisstock JC, Gore MG, Bottomley SP (1999). Probing the unfolding pathway of alpha1-antitrypsin. J Biol Chem.

[63] Cabrita LD, Dai W, Bottomley SP (2004). Different conformational changes within the F-helix occur during serpin folding, polymerization and proteinase inhibition. Biochemistry.

[64] Pace CN (1986). Determination and analysis of urea and guanidine hydrochloride denaturation curves. Methods Enzymol.

[65] Cabrita LD, Dai W, Bottomley SP (2006). A family of E. coli expression vectors for laboratory scale and high throughput soluble protein production. BMC Biotechnol.

[66] Haq, I. *et al*. Reactive centre loop mutants of alpha-1-antitrypsin reveal position-specific effects on intermediate formation along the polymerization pathway. *Biosci Rep* **33**, 10.1042/BSR20130038 (2013). 10.1042/BSR20130038 PMC3691886 23659468

[67] Calugaru SV, Swanson R, Olson ST (2001). The pH dependence of serpin-proteinase complex dissociation reveals a mechanism of complex stabilization involving inactive and active conformational states of the proteinase which are perturbable by calcium. J Biol Chem.

[68] Stone SR, Dennis S, Hofsteenge J (1989). Quantitative evaluation of the contribution of ionic interactions to the formation of the thrombin-hirudin complex. Biochemistry.

[69] Knaupp AS (2013). The roles of helix I and strand 5A in the folding, function and misfolding of alpha1-antitrypsin. PLoS One.

[70] Kumar S, Tamura K, Nei M (2004). MEGA3: Integrated software for Molecular Evolutionary Genetics Analysis and sequence alignment. Brief Bioinform.

[71] Krissinel E, Henrick K (2004). Secondary-structure matching (SSM), a new tool for fast protein structure alignment in three dimensions. Acta Crystallogr D Biol Crystallogr.

