# Supplementary material for: Probing the folding pathway of a consensus serpin using single tryptophan mutants
Source: Sci Rep. 2018 Feb 1;8:2121. doi: 10.1038/s41598-018-19567-9 (PMC5794792; doi:10.1038/s41598-018-19567-9)
Supplement: Supplementary file 1 — Supplementary Information [file 41598_2018_19567_MOESM1_ESM.pdf]

Probing the folding pathway of a consensus serpin using single tryptophan mutants

Li Yang, James A. Irving, Weiwen Dai, Marie-Isabel. Aguilar and Stephen P. Bottomley

Supplementary Information

Figures

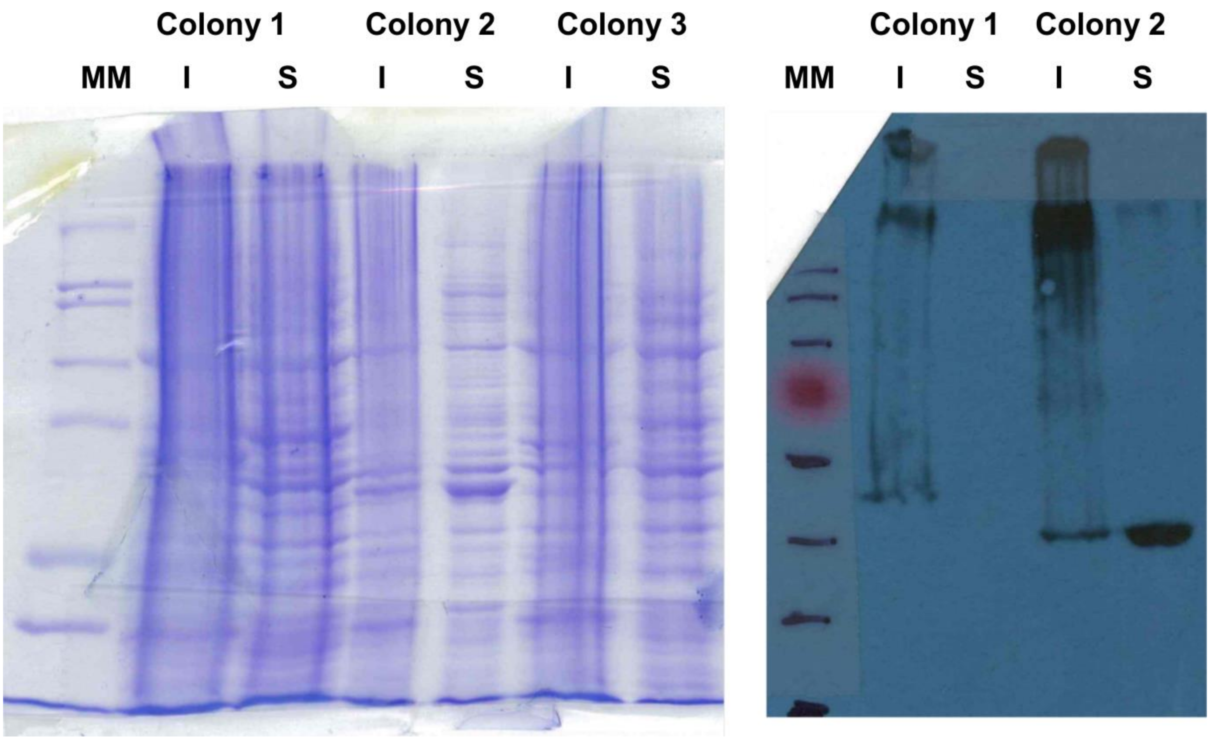

Fig. S1. Uncropped test expression gels for cAT. *Left:* small scale test expressions for selected *E. coli* colonies were resolved by SDS-PAGE. *Right*: the corresponding Western blot for colonies 1 and 2. MM shows the position of the molecular mass standard; I and S are insoluble and soluble fractions, respectively.
